# Application of a 3D volumetric display for radiation therapy treatment planning I: quality assurance procedures

**DOI:** 10.1120/jacmp.v10i3.2900

**Published:** 2009-07-17

**Authors:** Xing Gong, Mike Kirk, Tom Zusag, Gocha Khelashvili, James Chu, Josh Napoli, Sandy Stutsman

**Affiliations:** ^1^ Department of Radiation Oncology Rush University Medical Center Chicago IL 60612; ^2^ Actuality Medical Systems Bedford MA 01730 USA

**Keywords:** Three‐dimensional displays, treatment planning, quality assurance

## Abstract

To design and implement a set of quality assurance tests for an innovative 3D volumetric display for radiation treatment planning applications. A genuine 3D display (Perspecta Spatial 3D, Actuality‐Systems Inc., Bedford, MA) has been integrated with the Pinnacle TPS (Philips Medical Systems, Madison WI), for treatment planning. The Perspecta 3D display renders a 25 cm diameter volume that is viewable from any side, floating within a translucent dome. In addition to displaying all 3D data exported from Pinnacle, the system provides a 3D mouse to define beam angles and apertures and to measure distance. The focus of this work is the design and implementation of a quality assurance program for 3D displays and specific 3D planning issues as guided by AAPM Task Group Report 53. A series of acceptance and quality assurance tests have been designed to evaluate the accuracy of CT images, contours, beams, and dose distributions as displayed on Perspecta. Three‐dimensional matrices, rulers and phantoms with known spatial dimensions were used to check Perspecta's absolute spatial accuracy. In addition, a system of tests was designed to confirm Perspecta's ability to import and display Pinnacle data consistently. CT scans of phantoms were used to confirm beam field size, divergence, and gantry and couch angular accuracy as displayed on Perspecta. Beam angles were verified through Cartesian coordinate system measurements and by CT scans of phantoms rotated at known angles. Beams designed on Perspecta were exported to Pinnacle and checked for accuracy. Dose at sampled points were checked for consistency with Pinnacle and agreed within 1% or 1 mm. All data exported from Pinnacle to Perspecta was displayed consistently. The 3D spatial display of images, contours, and dose distributions were consistent with Pinnacle display. When measured by the 3D ruler, the distances between any two points calculated using Perspecta agreed with Pinnacle within the measurement error

PACS number: 07.07.Hj, 87.55.Qr, 87.56.Da, 87.55.D‐; Radiation Treatment Planning

## I. INTRODUCTION

The introduction of 3D treatment planning has significantly improved the quality of patient radiation therapy.^(^ ^1^ ^,^ ^2^ ^)^ Three‐dimensional treatment plans, based on patients’ CT or MRI images, provide better conformity and better target coverage than 2D treatment plans. In addition, dose conformity reduces the volume of normal tissue receiving high doses, ensuring that crucial organs are well protected from radiation exposure.

On conventional treatment planning systems, treatment planners normally see patients’ anatomy on 2D monitors slice‐by‐slice, or in three orthogonal planes: axial, sagittal, and coronal. An integrated 3D display device may alleviate this limitation and bring the treatment planning into an authentic 3D environment. Recently, many groups have been working on 3D displays in multiple fields, such as military, industry, research, medicine, oil and gas exploration, computer games and entertainment. Some of them apply holographic techniques; others adopt stereoscopic methods.^(^ ^3^ ^–^ ^9^ ^)^

The Perspecta System (Actuality Systems, Bedford, MA) is the first system that integrates volumetric 3D visualization with treatment planning to form a complete 3D planning system. The innovative volumetric display works by projecting a sequence of 2D patterns, or slices, onto a swiftly rotating omnidirectional diffuser screen in an enclosed Lexan dome. The Perspecta System is integrated with the Philips Pinnacle3 Treatment Planning System (v7.6c, Philips Medical Systems, Madison, WI), such that treatment plans can be easily transferred from Pinnacle to Perspecta for display and modification, and from Perspecta to Pinnacle for dose calculation. The Perspecta System is comprised of two parts, the PerspectaRAD RT Planning Workstation and the Perspecta Spatial 3D Display. The PerspectaRAD Workstation is synchronized with a Pinnacle Treatment Planning System (TPS) workstation and controls the display of images on the Spatial 3D Display. The 3D Display system allows the 3D visualization of the Pinnacle TPS objects such as the CT imageset, points‐of‐interest (POI), regions‐of‐interest (ROI), beams, and the Pinnacle calculated dose distribution. In addition, the PerspectaRad software and 3D mouse can modify the beam geometry (couch, gantry collimator angles) based on the visualization of the beams on the 3D display. The 3D designed beams can be transferred to the Pinnacle TPS for dose calculation. To assist the radiation oncologist during the review of treatment plans, the calculated dose distribution can then be rendered in the volumetric 3D display.

The synchronized two‐way communication between the TPS and the Perspecta System allows an iterative and efficient method of treatment planning. Since anatomical information is visible in a volumetric 3D display in a more natural and efficient way, treatment planners may create complex beam arrangements faster than with a 2D monitor screens. More importantly, the quality of treatment plans created using volumetric 3D visualization may be better than that based on 2D displays. A pilot study of 14 cases (12 brain, 1 lung, and 1 breast) has been designed and implemented to investigate these potentials of volumetric 3D visualization in treatment planning.^(10)^ The results demonstrate that better quality plans may be achieved with the use of 3D visualization compared to conventional 3D planning, with comparable planning efficiency. The lack of significant differences in planning efficiency may be related to the fact that volumetric 3D planning tools were not fully developed yet and the treatment planners were not as familiar with the operation of Perspecta's 3D system as the conventional TPS. An extended retrospective study with 33 patients (12 brain, 10 lung, 8 abdomen, and 3 pelvis) planned at three institutions reinforced the conclusion of the pilot study.^(11)^ In addition, physician evaluations of plans using 3D visualization were more efficient because all plan information (target coverage, normal tissue sparing, and the locations of hot or cold spots) from all CT slices were available simultaneously. Both the pilot and the extended studies indicate that volumetric 3D planning is a valuable contributor to cancer treatment with radiation.

3D planning systems require a rigorous quality assurance program, which is the major topic of this paper. The report of Task Group 53 of the Radiation Therapy Committee of the American Association of Physicists in Medicine (AAPM TG53) provides guidance on quality assurance of 3D treatment planning.^(12)^ Since the quality assurance of treatment planning systems is described elsewhere,^(13)^ the focus of this work is the design and implementation of quality assurance procedures for 3D display, specific 3D planning issues, and the integration with the Pinnacle TPS. The quality assurance tests proposed in the paper comply with the recommendations of AAPM TG53. However, the uniqueness of volumetric 3D planning leads to some alterations which will be discussed in the following sections. In this report, a design of a quality assurance program for a supplemental 3D display for a TPS is described; specifically, acceptance testing and quality assurance procedures to establish and monitor system performance.

## II. MATERIALS AND METHODS

The 3D planning system is an integration of a conventional 3D planning system (Pinnacle), and a volumetric 3D display (Perspecta). This paper focuses on the quality assurance of the latter. In this section, the details, features, and operation of the 3D display device (Perspecta Spatial 3D) and integration software (PerspectaRAD) are introduced, as well as supplementary materials. A set of acceptance tests are presented to guide the verification process of the 3D display based on the manufacture's guidelines and to determine the suitability of the 3D volumetric display for radiation treatment planning. A set of routine quality assurance tests, separated into hardware or software sections, have been developed. The hardware tests focus on the calibration of the Perspecta Spatial 3D Display with respect to manufacturer's specifications and the 3D mouse. The software tests focus on integrity of the data transferred displayed, including the CT imageset, POIs, ROIs, beams, and dose transferred from Pinnacle to Perspecta as well as beam data transferred from Perspecta to Pinnacle. Since the quality assurance procedures of the Pinnacle TPS have been described elsewhere, many of the tests use the Pinnacle TPS data as a benchmark to validate the PerspectaRad system.

### A. the Perspecta system

The Perspecta system is comprised of four parts: the Perspecta Spatial 3D display, 3D mouse, PerspectaRad workstation, and a set of Pinnacle HotScripts. These components when interfaced with a Pinnacle TPS allow radiation treatment planning in a 3D environment.

#### A.1 Perspecta spatial 3D display

Actuality Systems’ Perspecta Spatial 3D Display, version 1.9, was used. It projects floating, hologram‐like, color imagery into a 25 cm diameter sphere. The display is autostereoscopic and provides accurate depth cues from any angle. Due to glare and reflection off the spherical dome the images are most comfortably viewed with the room lights off or dimmed. The display has a 360 degree horizontal and 270 degree vertical field of view. The resolution is 768×768pixels per slice, with 198 slices intersecting a central vertical axis per revolution at a 30Hz volume refresh rate. A workstation (PerspectaRAD) connects to the display over a gigabit Ethernet link. The projected imagery can animate at interactive rates and the display is capable of magnification with no preset limit and rotates with 360 degrees rotation.^(14)^ In addition to the CT imageset, the Perspecta system displays points‐of‐interest (POIs), regions‐of‐interest (ROIs), beams, and dose.

#### A.2 3D mouse

A 3D haptic mouse, PHANTOM Omni, (SensAble Technologies, Inc., Woburn, MA), has been incorporated into Perspecta for 3D pointing. The 3D mouse is a multi‐function tool used to measure 3D distance, define end points for profile measurements, display dose at a point, and design beam orientation. New beam geometries and couch rotation can be quickly tried using a unique point‐click‐hold interface. Since the mouse must move in a 3D environment, the degrees of freedom must be 360 degrees of movement and have the ability to hold its position when the desired position has been located. The 3D mouse also functions as a 3D ruler.

#### A.3 PerspectaRAD RT planning workstation

PerspectaRAD RT Planning software (version 1.4.17100) controls the transfer of data from Pinnacle to Perspecta and Perspecta to Pinnacle, and provides a user interface to control objects (POIs, ROIs, beams, and dose) displayed in the Perspecta 3D Display. Additionally, the PerspectaRad interface allows the beam parameters (gantry, couch, collimator) to be modified.

#### A.4 Pinnacle HotScripts

The transfer of treatment planning data from Pinnacle to Perspecta and Perspecta to Pinnacle is handled by Pinnacle HotScripts (version 8.0m). The scripts rely on a combination of Unix and PERL scripts and internet file‐transfer‐protocol (ftp) software.

#### A.5 Phantoms

**Figure 1:**
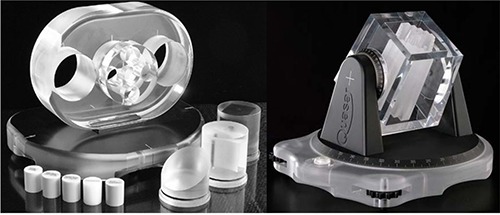
The Quasar Body Phantom (left) and MLC‐Phantom (right) used to test geometrical accuracy of data transferred from Pinnacle to Perspecta.

Two phantoms were used to perform the acceptance testing and quality assurance procedures. The QUASAR Multi‐Purpose quality assurance phantom system for advanced radiotherapy (Modus Medical Device Inc., London, Ontario, Canada) has been designed to perform both dosimetric and non‐dosimetric quality assurance tests for treatment planning recommended by AAPM Task Group #53 and Task Group #66.^(^ ^15^ ^,^ ^16^ ^)^ The main body of the phantom is an acrylic oval, with dimensions of $20\times 30\times 12\;{\text{cm}}^{3}$, shown in Fig. 1(left). Three cylinder openings with diameter of 8 cm can be filled with variety of inserts. The 27 cc acrylic cube within a 125 cc Delrin cube, and the 20° air wedge (40 cc) and 2 five‐cm‐long Delrin rods with diameters of 5 mm and 10 mm, respectively, were selected. The third cylinder opening was filled with a cedar cylinder simulating lung tissue. The geometric dimensions, volume, and CT number of the phantom and inserts were used to test the transfer of POIs and ROIs between Pinnacle and Perspecta.

The QUASAR Beam Geometry MLC phantom, shown in Fig. 1(right), was used. The phantom rotates about an isocenter, designated by a 1 mm diameter stainless steel sphere, on the vertical (couch) and horizontal (gantry) axes. The trapezoidal phantom body is narrower at the top and wider at the base and is designed to follow the beam divergence of a 100 SAD beam. The inner material forms a step‐wise object for beam aperture tests.

### B. Acceptance testing

The crucial components of treatment planning with supplemental 3D displays are the accurate transfer of data between the TPS and the 3D display and the accuracy of the 3D display. The acceptance testing process centers on the ability of the Perspecta system to accurately display a treatment plan generated on the Pinnacle TPS. The data transfer depends on the computer hardware and software functionality. A set of generalized tests, listed in Table 1, detailing the hardware and software processes to guide the acceptance testing process.

**Table 1:** Acceptance tests.

*Test*	*Method*	*Result*
*Hardware*		
3D Display	Check volume refresh rate and reduce image wobble by calibrating display.	>30Hz
3D Mouse	Check that mouse holds position and has full range of motion inside 3D display.	approx. 1 mm after calibration
3D Display Workstation	Check bi‐directional data transfer.	<1mm, 360° rotation
*Software*		
Image Input	Transfer and display a set of CT images of a phantom with known internal and external dimensions	Positional geometry and CT numbers consistent with TPS within 1%
Point‐of‐Interest Display	Transfer and display a ray of points and check consistent location, size, color	Consistent data and consistent with TPS within 0.1 cm
Region‐of‐Interest Display	Transfer and display set of ROIs and check for consistent location, size, color, volume	Consistent data and consistent with TPS within 0.1 cm
Beam Display	Transfer beams from Pinnacle to Perspecta and Perspecta to Pinnacle. Check consistent FS, color, SSD, and beam geometry (gantry, collimator, couch)	Consistent data and consistent with TPS within 0.1 cm and within 1°
Dose Display	Transfer dose from TPS to Perspecta and confirm consistent dose at points and volumes	1% and 1 mm

### C. Hardware acceptance

The hardware components including the 3D display and 3D mouse were inspected for baseline performance.

#### C.1 Perspecta 3D display calibration

The 3D image quality is dependent on a finely calibrated device. The Perspecta display works by overlapping a front‐projected volume and a rear‐projected volume. A slight mismatch in their calibration contributes to jitter or shake in the image. Each calibration plane, analogous to a single CT slice, is projected twice per rotation – once from the front and once from the back. The goal of the calibration process is to eliminate any double images caused by the misalignment of these two projections. Adjustments are made within a single plane at a time, then on each successive plane until all 12 planes have been calibrated. A well‐calibrated display has zero wobble in most of the image, with approximately 1 mm physical jitter (equivalent to the average voxel size) in areas that are simply unable to be calibrated completely due to manufacturing issues. Three factors which contribute to this situation are: “front‐scan” and “back‐scan” images that are not perfectly overlaid, slight manufacturing imperfection in the inner clear dome that houses the screen, and potentially a deviation from perfect rotational symmetry. The jitter causes a slight blurring of images and planning objects, which becomes increasing evident as the device moves out of calibration.

#### C.2 3D mouse

The 3D mouse was tested to ensure necessary functionality. The mouse's ability to navigate inside the spherical 3D display was checked. The mouse has the ability to select points in the display and measure the distance between them with sub‐millimeter precision. Since the 3D mouse moves in both a vertical and horizontal direction, it has the ability to be mechanically locked and held at a specific position without the user holding it.

### D. Software acceptance

Specific acceptance tests for each software acceptance test were designed and performed to provide the baseline performance standards. This critical baseline data is then checked as part of a comprehensive quality assurance program.

#### D.1 PerspectaRAD RT planning software

The ability of the PerspectaRad planning workstation to initiate and transfer data was tested. The PerspectaRad workstation sends and receives data from Pinnacle via ftp. In addition, the 2D workstation display provides a user interface for the control of Pinnacle treatment planning objects on the 3D display, including turning any of the objects on or off on the display. Any errors encountered during the transfer of data are stored in an error log file.

#### D.2 Image input

3D treatment planning is usually based on reformatted CT images of the patient's anatomy. Task Group #53 specifies steps to confirm the validity of reformatted images and the grayscale window and level settings of the display. The specific image input quality assurance tests are specified in Table 2.

**Table 2:** Acceptance: Image input

*Test*	*Method*	*Result*
Image Format CT Number	Consistency with Pinnacle reformatted images	1%
Scan Orientation	Accurate patient representation (Supine/Prone, Head/Feet First, Left/Right)	Pass
Imaging Table	Accurate removal of imaging table for radiation planning purposes	Pass
Image Warp/Distortion	Visual inspect of phantom imagesets and linear arrays	Pass
Spatial Consistency	Measure distance between CT landmarks	1 mm
Window/Level	Ability to vary window and level over the range of typical CT values	Pass

##### D.2.1 Image format and CT number

Similar to Pinnacle, Perspecta reformats the original CT images before displaying; as a result, tests are recommended to verify the consistency between the new images and the original CT images. Since only the primary imageset is viewable in Perspecta, the tests are limited to the CT dataset. To implement the tests, a CT scan of the QUASAR body phantom was used. Using the Pinnacle TPS, three pairs of POIs were created and CT number profiles as a function of distance between the POIs were calculated. Three profiles representing the CT numbers along anterior‐posterior, left‐right, and superior‐inferior directions were created. Using the 3D mouse, profiles between the same points were obtained on Perspecta. A comparison of the Pinnacle and Perspecta CT number profiles provided a quantitative assessment of the consistency between the Pinnacle and Perspecta imagesets. Since there is no American National Standards Institute (ANSI) standard for measuring contrast in a volumetric 3D display and the brightness specification was not provided by the manufacturer, individual users must qualitatively determine if the display image has degraded beyond acceptable levels.

##### D.2.2 Scan orientation

The patient CT scan orientation during the CT scan is variable, thus the TPS must accurately display the patient position during the treatment planning process. Three CT scans with different patient orientations, listed in Table 3, were imported into Pinnacle and displayed on Perspecta. The central axis of two perpendicular beams with gantry angle of 0° and 270° were displayed on Perspecta as well. The relationship between the patient orientation and orthogonal beams was checked for consistency.

**Table 3:** 

*Site*	*Brain*	*Breast*	*Extremity*
Scan Orientation	Supine, Head First	Prone, Head First	Supine, Feet First

##### D.2.3 Imaging table

During treatment planning, the imaging table must be removed for accurate dose calculation and treatment delivery. The Perspecta 3D planning system relies on the table as determined in Pinnacle. The portion of the image below the couch removal line is not displayed in Perspecta. To verify the accuracy of this function, the QUASAR body phantom was used. The distance between the top of the phantom and the couch removal line was checked for consistency between Pinnacle and Perspecta.

##### D.2.4 Image warp / distortion

The 3D display modification tools, such as magnification and rotation, were tested to determine any deleterious effects on the integrity of the imageset. Additionally, linear edges of the body phantom were used to check for image warping.

##### D.2.5 Spatial consistency

The distance between different points in the CT imageset was calculated using the 3D Ruler functionality of the 3D mouse to validate the spatial integrity of the images. CT markers were placed on the QUASAR body phantom. The distance as measured on Pinnacle was used to check the 3D mouse ruler functionality on Perspecta. The CT imageset was magnified to arbitrary levels on Perspecta and Pinnacle.

##### D.2.6 Window / Level

The ability to vary grayscale color associated with various CT numbers, known as window and level, is important in aiding the identification of internal organs and lesions. The window and level settings are adjusted in the software using a sliding tool bar. It is not possible to specify specific numerical values. To test the ability of Perspecta to assign the grey color, the QUASAR CT phantom with various cylindrical inserts of specific electron densities was imported. For each insert, a small window value was created around a level corresponding to the approximate mean CT number of the internal object. The 3D display was qualitatively inspected for agreement with the Pinnacle 2D display.

### D.3 Point of interest display

The accurate location of POIs on Perspecta is crucial for beam alignment during treatment planning. In addition, the name, color, and x‐y‐z coordinates of the points should be consistent and displayed accurately. The 3D point array was used to check the transfer of POIs to Perspecta. The array of points was also used to check display warping on the periphery of the display. The specific POI quality assurance tests are specified in Table 4.

**Table 4:** Acceptance: Point and region‐of‐interest.

*Test*	*Method*	*Result*
Consistency Tests	POI and ROI display characteristics consistency	Pass
	POI spatial accuracy	1 mm
	POI/ROI and image consistency	1 mm
ROI 3D Display Features	ROI volume consistency Surface rendering	1% Pass
	Multiple ROIs per slice	Pass
	Ring ROIs	Pass
	Capping	Pass

#### D.3.1 POI display characteristics consistency

The name and color of POIs transferred from Pinnacle to Perspecta was checked for consistency.

#### D.3.2 POI spatial consistency

POI locations were checked for consistency in the center and the periphery of the spherical display. Two $5\times 5\times 5\;{\text{cm}}^{3}$ point arrays were created to cover both the central and peripheral area of Perspecta. The three‐dimensional point array consists of 125 POIs separated by 1 cm. The 3D mouse on Perspecta was used to measure the distances between pairs of POIs in the point array. Seven pairs of POIs were chosen randomly and distances between them as calculated on Pinnacle and Perspecta were compared. In addition, a visual inspection of the 3D point arrays was performed to check the linearity of the POIs by viewing their overlap along lines intersecting them.

#### D.3.3 POI and image consistency

POIs were placed at specific points on the CT imageset based on radio‐opaque CT marks placed during the CT scan. The alignment of the POIs with the CT markers was checked on Perspecta.

### D.4 Region of interest display

ROIs appear as 3D wireframe contours superimposed on CT‐based anatomy in Perspecta. The accuracy of ROI shape and location relative to CT anatomy was checked. Specific ROI quality assurance tests are specified in Table 4.

#### D.4.1 ROI display characteristics consistency

The accurate transfer of specific ROI name and color from Pinnacle to Perspecta was checked.

#### D.4.2 ROI and image display consistency

To verify the accuracy of the contour display with respect to the image display, the QUASAR body phantom was used. The geometric accuracy of the ROIs was investigated based on the correlation between the ROIs and the internal phantom objects. On Perspecta, the superposition of each contour was visually identified with its associated 3D structure and provided a qualitative verification of the ROI display registration with the image display. In addition, since the phantom objects are made of different materials and therefore represented by different CT numbers, the CT number variation can be used to localize the object position. For example, the left‐right CT number profile taken from the QUASAR body phantom passed through the cedar cylinder, Delrin cube, Acrylic cube, 5 mm Delrin rod, air wedge, and 10 cm Delrin rod. The increase or decrease in the CT numbers as a function of distance was used to localize each of the objects. The ROI borders which are denoted as vertical lines superimposed on a CT number profile were checked for agreement.

#### D.4.3 ROI volume accuracy

A validation of the ROI volume in PerspectaRad was performed by contouring multiple objects in the QUASAR body phantom. The Pinnacle and Perspecta calculated volumes were compared with the expected volumes. Density overrides specified in Pinnacle are not transferred to Perspecta.

#### D.4.4 ROI 3D rendering

The Perspecta display's ability to render the exterior of ROIs was investigated. In addition, the ability to display unusual ROIs, such as multiple contours belonging to a single ROI on a single slice (bifurcated contours) and ring or donut ROI's, was checked. The end effect – or capping – of ROIs was also checked in Perspecta.

### D.5 Beam display

The geometric accuracy of the isocenter and beam geometry is essential for treatment planning and delivery. The 3D visualization of complex beam orientations with couch rotations using a 3D volumetric display provides an advantage over conventional planning systems using 2D displays. In Perspecta, the beam central axis is displayed as well as the beam apertures as designed in Pinnacle. Treatment accessories such as wedges, bolus, and compensators are not displayed. The beam apertures for a single control point (segment) are viewable on Perspecta. Specific beam display quality assurance tests are specified in Table 5.

**Table 5:** Acceptance: Beams.

*Test*	*Method*	*Result*
Consistency Tests	Beam data	Pass
	Gantry angle	1°
	Collimator angle	1°
	Couch angle	1°
Beam Display	Geometric accuracy	1 mm
	Divergence	1 mm
	Block aperture	Pass

#### D.5.1 Beam Data

Treatment beam data, unlike POIs and ROIs, can be transferred from Pinnacle to Perspecta and from Perspecta to Pinnacle. Specific beam parameters such as name, color, gantry, collimator, and couch rotations were checked for consistency between the two systems after data transfer.

#### D.5.2 Beam geometrical accuracy

The geometric accuracy of the treatment beams was tested in two ways. First, using the $5\times 5\times 5\;{\text{cm}}^{3}$ point array, ten different beams were generated whose central axes passed through the center of either three or five POIs in the array. The specific gantry and couch angles are detailed in Table 6. The points that the beams should intersect were noted and verified on Perspecta. In addition, the MLC phantom was configured to be aligned with seven different couch and gantry angles, as detailed in Table 7, and CT scanned. Seven beams with the same gantry angles and couch angles were generated and displayed in Perspecta. The central axis at each couch and gantry angle of rotation was checked for its alignment with the center axis of the phantom body.

**Table 6:** Gantry and couch angles for beam‐point array test.

	*Beam*									
	*1*	*2*	*3*	*4*	*5*	*6*	*7*	*8*	*9*	*10*
Gantry Angle	0	90	270	90	0	45	315	63	333	64
Couch Angle	0	0	270	315	297	325	305	90	48	66

**Table 7:** Gantry and couch angles for MLC phantom tests.

	*1*	*2*	*3*	*4*	*5*	*6*	*7*
Gantry Angle	90	0	0	90	60	48	290
Couch Angle	0	0	90	90	30	348	290

#### D.5.3 Beam divergence and aperture

In addition to the accuracy of the beam's central axis, the beam's geometric divergence as displayed on Perspecta was checked. The QUASAR MLC phantom was also used to test the accuracy of the beam divergence and aperture as displayed on Perspecta. Five isocenters were set on Perspecta to check the beam divergence from 80 to 130 cm SSD using the mlc phantom. For a 10 cm square field defined at isocentre, the divergent field width was checked at 80, 95, 105, 130 cm SPD. On Perspecta, beams with field sizes of 1×2cm,5×5cm, and 10×10cm were created to test their alignment with the center, along with the inner and outer portions of the phantom body. The MLC aperture was tested using the MLC phantom. In Pinnacle, the MLC (or block) edges of the aperture was aligned to the staircase MLC apertures, and the beam was transferred to Perspecta where the beam aperture was checked for agreement with the MLC phantom image. The beam aperture cannot be modified using the PerspectaRad software.

### D.6 Dose display

An accurate representation of the dose calculation is crucial for treatment planning and evaluation. When shown on the 3D display, tumor target coverage, normal tissue sparing, and hot and cold spots can be detected at a glance. The specific dose display quality assurance tests are detailed in Table 8.

**Table 8:** Acceptance: Dose display.

*Test*	*Method*	*Result*
Consistency Tests	Dose at point	1%/1 mm
	Rotation and tilt	Pass
	Dose profile	1%/1 mm
Dose Display	Dose grid	1%/1 mm
	Isodose surfaces	1%/1 mm

#### D.6.1 Dose points

To investigate the accuracy of 3D dose display, the 5×5×5cm point array was used. The default dose grid was $0.4\times 0.4\times 0.4\;{\text{cm}}^{3}$. Using the 3D mouse, the dose at any point is displayed on the PerspectaRAD workstation. Ten points were randomly chosen for comparing the dose read from PerspecaRAD to those doses from Pinnacle at same points. Because the point positions were accessed by the 3D mouse, it was also an interactive way to check the dose at specific points, as recommended by AAPM Task Group #53.

#### D.6.2 Dose consistency with rotation

The 3D image in Perspecta can be rotated around or tilted up/down. It is necessary to check the consistency of the display after rotation or tilting. The display was rotated and tilted to an arbitrary angle. The shape and orientation of the rotated dose distribution were examined to confirm consistency with the original dose distribution.

#### D.6.3 Dose grid

AAPM Task Group #53 suggests verifying the dose interpolation functionality with both small and larger dose grid spacing. At default, the dose grid resolution on Pinnacle is 0.4 cm. Two other resolutions, 0.3 cm and 0.5 cm, were selected for this test. For each resolution, the following steps were executed: the resolution of the dose grid was set, the dose was computed on Pinnacle, and was then transferred to Perspecta where a dose profile between the two points was calculated. The dose profiles were compared with those from Pinnacle.

## E. Quality assurance

Based on the baseline performance data during acceptance testing, quality assurance testing procedures and frequency were designed to confirm that the Perspecta spatial 3D display was operating within its tolerance limits. A subset of the acceptance tests are proposed for routine quality assurance.

## III. RESULTS & DISCUSSION

In this section, the results of the Perspecta display acceptance tests are presented. The consistency of the CT data and planning system structures (POIs, ROIs, beams, and dose) as displayed by Perspecta were compared with the known values from the QUASAR phantoms and the Pinnacle TPS.

### A. Acceptance testing

A quantitative assessment of the ability of Perspecta to participate in the treatment planning process based on the transfer of data between Perspective and Pinnacle was performed. The general acceptance tests listed in Table 1 were performed to confirm that the Perspecta system is capable of transferring treatment planning data between Pinnacle to Perspecta based on a qualitative assessment of data consistency. The results of the specific quantitative tests of data consistency are outlined below.

### B. Hardware acceptance

The functionality of the 3D display, 3D mouse and PerspectaRad workstation were tested. The results of those tests are detailed below.

#### B.1 Perspecta 3D display calibration

The calibration procedure decreased the image wobble visible to the eye to approximately 1 mm and the volumetric refresh rate is sufficient to discern anatomical detail on the CT images set.

#### B.2 3D mouse

The 3D mouse held its position when using the lock‐and‐hold function and provided access to the entire 3D spherical Perspecta display. The mouse‐specific points inside the imageset could be selected for dose measurements or designating the endpoints of profile measurements.

## C. Software acceptance

The results of the tests documenting the consistency of data transferred between Pinnacle to Perspecta.

### C.1 PerspectaRad software

The PerspectaRad software received data from Pinnacle and initiated data transfer to Pinnacle. The Pinnacle HotScripts initiated data transfer to PerspectaRad and received data from PerspectaRad. The software tests detail the accuracy of the data transferred.

### C.2 Image input

#### C.2.1 Image format and CT number

**Figure 2:**
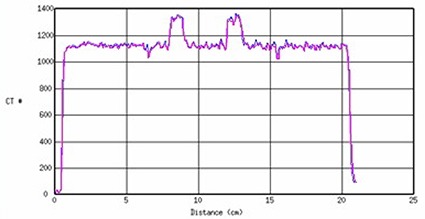
The CT number profiles along a posterior‐anterior line for Pinnacle (blue) and Perspecta (pink). The horizontal axis is distance (cm) and vertical axis is CT number.

The consistency of the CT imageset as displayed on Perspecta with Pinnacle's reformatted CT imageset was used to validate Perspecta's 3D display. Three CT number profiles from Pinnacle and Perspecta representing the CT numbers along anterior‐posterior, left‐right, and superior‐inferior direction of the body phantom were calculated. The overlap between the Pinnacle and Perspecta CT number profiles in the posterior‐anterior line is shown in Fig. 2 and was in agreement within 1% or 1 mm.

#### C.2.2 Scan orientation

**Figure 3:**
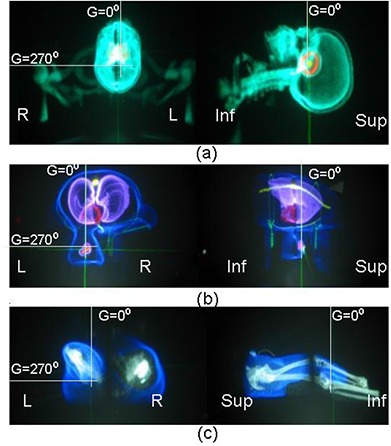
The images show an axial and sagittal view of (a) a head‐first scan of a supine patient, (b) a head‐first scan of a prone patient, and (c) a feet‐first scan of a supine patient. An AP (0 degree) and lateral (270 degree) beams are denoted by the perpendicular white lines in Figs. 3(a–c). In each case, a transverse view is presented from the end of couch (opposite to gantry) and a sagittal view with an inferior (Inf) to superior (Sup) label. The patient's left (L) and right (R) are denoted.

The Perspecta device's ability to maintain the correct scan orientation was tested using beams with known gantry angles. Three cases with different patient positioning during the CT scan were loaded into Perspecta to test the scan position with respect to beam angles. These included a head‐first scan of a supine patient, a head‐first scan of a prone patient, and a feet‐first scan of a supine patient. Both an AP (0 degree) and lateral (270 degree) beams were created and denoted by the perpendicular white lines in Figs. 3(a)–3(c). In each case, a transverse view is presented from the end of couch (opposite to gantry) and a sagittal view with an inferior (Inf) to superior (Sup) label. The labels were added for clarity and are not part of the Perspecta display. The patient scan orientation as judged by the beams‐eye view of orthogonal films is consistent as displayed on Perspecta.

#### C.2.3 Imaging table removal

The respective distances between the top of the table and the top of the phantom were measured on Pinnacle and Perspecta and agreed within 1 mm. The distance from the top of the phantom to the couch was measured to be 18.3 cm on Pinnacle and 18.3 cm on Perspecta. Since the Perspecta system relies on Pinnacle for dose computation and does not perform dose calculation, the accuracy of imaging table removal is for display purpose only.

#### C.2.4 Image warp and distortion

The display was tested to ensure that different magnitudes of magnification and 3D rotation did not induce any distortion or warping visible to the eye.

#### C.2.5 Spatial consistency

The distance between landmarks denoted by CT markers on the QUASAR phantom CT scan were measured on Pinnacle and Perspecta using the 3D mouse. The agreement between measurements was within 1 mm – which is acceptable give the uncertainty in selecting the consistent point between the two displays using the conventional mouse on Pinnacle and 3D mouse on Perspecta.

#### C.2.6 Window / Level

The window and level selection was acceptable for treatment planning purposes. The settings were comprehensive enough to match preselected display settings typically found in TPS such as bone, lung, breast, and abdomen settings found in Pinnacle. However, the specific values were not specified.

## C.3 POI display

### C.3.1 POI data consistency

The POI name and color were correctly transferred from Pinnacle to Perspecta.

### C.3.2 POI spatial consistency

**Figure 4:**
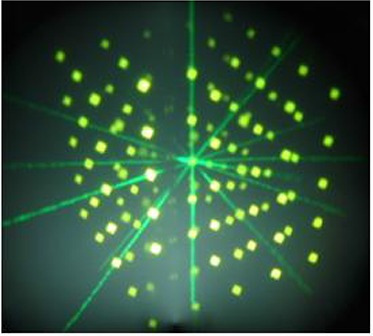
The 5×5×5 point array as displayed on Perspecta. The distance between the points in the x,y and z directions is 1 cm.

The distance between pairs of POIs was compared between Pinnacle and Perspecta. The agreement between the Pinnacle and Perspecta measured distances, in addition to the calculated distance based on the POI location inside the array, is shown in Table 9. The 5×5×5 point array as displayed on Perspecta is shown in Fig. 4. The distance between seven randomly selected pair of points was measured. The agreement was within 1 mm.

**Table 9:** Comparisons of ruler measurements in Perspecta and Pinnacle.

	*Distance (cm)*						
	*#1*	*#2*	*#3*	*#4*	*#5*	*#6*	*#7*
Calculated	6.93	4.58	5.39	4.7	4.36	3.0	3.32
Pinnacle	6.93	4.58	5.39	4.69	4.36	3.0	3.32
Perspecta	6.9	4.6	5.4	4.7	4.4	3.0	3.3

### C.3.3 POI and image consistency

The POI transferred to the Perspecta display aligned with the corresponding CT markers it was aligned with on Pinnacle within 1 mm. This verifies that the correlation between the POI and the CT imageset was maintained during the data transfer.

## C.4 ROI display

### C.4.1 ROI data

The Pinnacle ROIs were accurately transferred from Pinnacle to Perspecta with the corresponding name and colors.

### C.4.2 ROI and image display consistency

**Figure 5:**
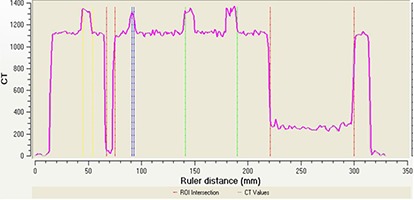
A CT number profile as a function of distance along a line passing through the contours. The increase and decrease in CT number denotes the edges of the internal structures within the body phantom. The colored vertical lines demark the edge of the ROIs as drawn in Pinnacle along the same line, while the pink solid line represents the CT number profile.

The consistency of the ROIs location within the CT imageset after transfer to the Perspecta 3D display was verified. A CT number profile as a function of distance along a line passing through the contours is shown in Fig. 5. The increase and decrease in CT number denotes the edges of the internal structures within the body phantom. The colored vertical lines demark the edge of the ROIs as drawn in Pinnacle along the same line, while the pink solid line represents the CT number profile. This profile provides a quantitative measure of the agreement. There is a small shift of the ROI edges with respect to the center of the rising or dropping of CT number profile. The shifts are less than 2 pixels (1 mm) in this case. The reason for this displacement is attributed to the manual contouring of ROIs in Pinnacle, which has slight variation depending on the window and level settings chosen.

### C.4.3 ROI volume accuracy

The volume of the ROIs as calculated by Pinnacle and PerspectaRad were compared with the expected (manufacturer supplied) volumes. The volumes are listed in Table 10. The differences between the volumes from Pinnacle or Perspecta and the known volumes inside the phantom are larger than the differences between the Pinnacle and Perspecta calculated volumes. Since the CT slice thickness is a finite value (3 mm), the calculated volume will differ from the actual value.^(17)^ These errors are introduced by the manual contouring process. Thus for this comparison, the focus is on the difference between Pinnacle and Perspecta calculated volumes. According to both CT studies, the volumes calculated by Perspecta and Pinnacle are within 1%, except for the 5 mm rod, whose entire volume is less than 1 cc.

**Table 10:** Comparison of ROI volumes.

*ROI*	*Volume* $\left({\text{cm}}^{3}\right)$			*% Difference*
	*Expected*	*Pinnacle*	*Perspecta*	*Pinnacle vs. Perspecta*
Air Wedge	40.0	42.3	42.2	0.2
Cedar Cylinder	603.2	610.1	610.0	0.0
Delrin Cubes	125.0	123.7	123.6	0.1
5 mm Rod	1.0	0.9	0.9	1.0
10 mm Rod	3.9	3.7	3.7	0.0

### C.4.4 ROI 3D rendering

ROIs are displayed in the Perspecta as a series of wireframe slices. The last slice of the ROI formed the end or cap of the volume. Ring (or donut) ROIs are displayed in Perspecta; in addition, ROIs consisting of two independent ROIs on a single slice can also be displayed. Since non‐axial ROI can not be drawn in Pinnacle using the paintbrush tool, the ability of Perspecta to display these ROIs was not evaluated.

## C.5 Beam display

Unlike the POI, ROI and dose display tests, the verification of consistent beam data involved both data transferred from Pinnacle to Perspecta and Perspecta to Pinnacle.

### C.5.1 Beam data

The beam data (name, color, gantry, collimator, and couch angles, SSD) were accurately transferred from Pinnacle to Perspecta and Perspecta to Pinnacle. However, the linear accelerator machine limits are not enforced within the PerspectaRad software; thus Pinnacle will display an error and reset the parameter if it was positioned beyond its limit in PerspectaRad.

### C.5.2 Beam geometric accuracy

Ten beam angles were designed to test the beam geometric accuracy using the $5\times 5\times 5\;{\text{cm}}^{3}$ point array. The center point of the array was chosen to be the isocenter. The axes of all 10 beams should intersect three or five of the POIs making up the point array, depending on their couch and gantry angles. As shown in Fig. 4, the axes of the 10 beams, with gantry and couch angles listed in Table 6, were visually inspected to pass through the isocenter (within 1 mm) and the POIs in the array. The last three combinations of gantry and couch angle were for non‐axial beam angles.

### C.5.3 Beam divergence and beam aperture

The QUASAR phantom was scanned, corresponding to the various combinations of gantry and couch angles listed in Table 7. The beams‐eye view (BEV) of an axial beam configured with the same gantry and couch rotation was visually inspected to intersect with the top of the phantom body, and the beam divergence matched the divergence of the phantom body within 1 mm. The SPD equals 100 cm, indicating the point is at the isocenter. At various SPD values (80, 95, 100, 105 and 130) the beam field size was measured. The measured error on Perspecta was less than 1 mm. The accuracy of the beam divergence was verified using the QUASAR MLC phantom scans used for beam geometric accuracy.

## C.6 Dose display

### C.6.1 Dose points

The 5×5×5 point array has been applied to test the ability to measure the point dose on Pinnacle and Perspecta. As a consistency check, the dose was compared at nine points in Perspecta and Pinnacle. The dose at all points was within 1%.

### C.6.2 Dose consistency with rotation

By rotating the dose distribution around on Perspecta, it was visually observed that the dose distribution rotates without distortion. Doses at specific points were sampled and were invariant to rotation.

### C.6.3 Dose grid

**Figure 6:**
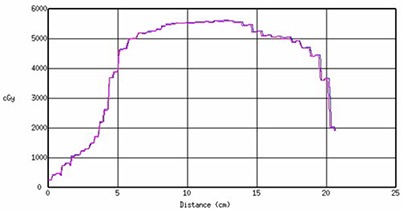
The dose profiles for Pinnacle (blue) and Perspecta (pink). The horizontal axis is distance (cm) and vertical axis is dose in cGy.

To evaluate the interpolation of dose grids with different spacing, we made profiles along a chosen line, crossing the dose distribution from both Pinnacle and Perspecta with 3 mm, 4 mm, and 5 mm dose grid resolutions. The dose profile for a 4 mm grid size is shown in Fig. 6 where the dose profiles from Pinnacle (blue) and Perspecta (pink) are shown. The profiles from Pinnacle and Perspecta agree with each other within 1% and 1 mm of the dose displayed in Pinnacle for all dose grid resolutions tested.

### D. Quality assurance frequency

Specific tests should be performed to assure users that system performance has not deteriorated. A calibration of the 3D display system may be necessary when the user observes excessive image shake or jitter. The need to recalibrate increased with increased use of the device. Since our device sat on a wheeled cart and was moved about the office often, a monthly calibration was necessary. Users noticed a significant improvement in the display clarity after calibration. The full set of software acceptance tests should be performed for each new version of the Perspecta or Pinnacle software to ensure accurate data transfer. The interval for specific quality assurance tests are detailed in Table 11.

**Table 11:** Suggested quality assurance frequency.

*Frequency*	*Task/Test*	*Action/Tolerance*
Daily	Check error log	Fix cause of error
Monthly	3D display calibration	Perform calibration
Software Upgrade	Software acceptance tests	Confirm accurate transfer

## IV. CONCLUSIONS

Recent advances in computer and engineering technologies have stimulated tremendous development in 3D stereoscopic and virtual reality systems.^(18)^ Being a heavily imaging‐focused medical discipline, these technologies present excellent opportunities for enhancing the practice of radiation oncology. Although current research activities in this area are largely in radiotherapy treatment planning, plan evaluation, and training and education, these technologies also have potential for improving our ability to deliver image guided treatment interventions.^(^ ^19^ ^–^ ^21^ ^)^ As increasing precision is expected for modern radiation treatment planning and delivery, it is necessary to ensure that these promising visualization technologies are displaying the image and dose data accurately. The quality assurance issues related to computer displays are addressed in AAPM Radiation Therapy Committee Task Group 53. The current study illustrates how these principles can be applied to new visualization technology.

The data demonstrate that the 3D visualizing Perspecta device has been successfully integrated with a conventional 3D treatment planning system. The 3D spatial display of images, POIs, ROIs, and dose distributions are accurate. The 3D mouse can also be used to properly define beam angles and apertures. However, the Perspecta display's accompanying software, PerspectaRad, does not enforce machine limits. The ability to commission the display to the user's specific machine limitation would benefit treatment planning with the 3D display. Although this visualization technology has the potential to speed up and/or improve the treatment planning process, a more seamlessly integrated system would be required to fully realize the potential benefit. These findings warrant the continued development of this technology for radiation oncology applications.

## ACKNOWLEDGEMENTS

The authors wish to thank the anonymous referees whose suggestions strengthened this work.

## References

[1] Perez C A , Purdy J , Harms W. et al. Three‐dimensional treatment planning and conformal radiation therapy: preliminary evaluation. Radiother Oncol. 1995;36(1):32–43. 8525023 10.1016/0167-8140(95)01566-y

[2] Weigel T , Schmidt R , Krüll A. , Schwartz R , Sommer K , Hübener KH . Advantage of three‐dimensional treatment planning for localized radiotherapy of early stage prostatic cancer. Strahlenther Onkol. 1992;168(12):692–97. 1481118

[3] Haller M , Holm R , Volkert J , Wagner R . A VR based safety training in a petroleum refinery. In: Eurographics 99. Proc. of European Association for Computer Graphics; 1999 Sept. 7–11; Milan, Italy. Aire‐la‐Ville (Switzerland): EACG; 1999.

[4] Hubbold RJ , Hancock DJ , Moore CJ . Autostereoscopic display for radiotherapy planning. In: Proceedings electronic imaging '97 symposium. Conference on stereoscopic displays and virtual reality systems IV. Volume 3012 Virginia (NC): Society for Imaging Science and Technology; 1997.

[5] Isambert A. , Beaudré A , Ferreira I , Lefkopoulos D . Quality assurance of a virtual simulation software: application to IMAgo and SIMAgo (ISOgray). Cancer Radiother. 2007;11(4):178–87. 17418608 10.1016/j.canrad.2007.03.001

[6] Able, CM and Thomas MD . Quality assurance: fundamental reproducibility tests for 3‐D treatment‐planning systems. J App C Med Phys. 2005;6(3):13–22. 10.1120/jacmp.v6i3.1983 PMC5723495 16143788

[7] Hubbold RJ , Hancock DJ . Stereo display of nested 3d volume data using automatic tunneling. In: Stereoscopic Displays and Virtual Reality Systems VI. Proc. of the SPIE Volume 3639. San Jose (CA): The International Society for Optical Engineering; 1999 p. 200–207.

[8] Lee JS , Jani AB , Pelizzari CA , et al. Volumetric visualization of head and neck CT data for treatment planning. Int J Radiat Oncol Biol Phys. 1999;44(3):693–703. 10348301 10.1016/s0360-3016(99)00042-5

[9] Patel D , Muren LP , Mehus A , Kvinnsland Y , Ulvang DM , Villanger KP . A virtual reality solution for evaluation of radiotherapy plans. Radiother Oncl. 2007;82(2):218–21. 10.1016/j.radonc.2006.11.024 17224194

[10] Chu, JC , Gong X , Kirk M , et al. Holographic image guided radiation therapy (HIGRT) treatment planning: a Multi‐Institutional Study [Abstract]. Int J Radiat Oncol Biol Phys. 2006;66(3):6664–65.

[11] Chu JCH , Gong X , Cai C. et al. Multi‐institutional randomized study to evaluate a holographic display device for treatment planning. Accepted at ASTRO 2007 Oct 28–Nov 1; Los Angeles, California: Am Soc for Therapeutic Radiology & Oncology; 2007.

[12] Fraass B , Doppke K , Hunt M. , et al. Quality Assurance for Clinical Radiotherapy Treatment Planning. AAPM Radiation Therapy Committee Task Group 53. Med Phys. 1998;.25(10):1773–1829. 9800687 10.1118/1.598373

[13] Kutcher GJ , Coia L , Gillin M , et al. Comprehensive QA for radiation oncology: report of AAPM Radiation Therapy Committee Task Group 40. Med Phys. 1994;21(4):581–618. 8058027 10.1118/1.597316

[14] G. E. Favalora , J. Napoli , D. M. Hall , et al. 100 Million‐voxel volumetric display. In: Hopper Darrel G., editor. Cockpit Displays IX: Displays for Defense Applications. Proc. of SPIE Vol. 4712 SPIE; 2002 p. 300–12.

[15] Mutic S , Palta JR , Butker EK , et al. Quality assurance for computed‐tomography simulators and the computed‐tomography‐simulation process: report of the AAPM Radiation Therapy Committee Task Group No. 66. Med Phys. 2003;30(10):2762–92. 14596315 10.1118/1.1609271

[16] Craig T , Brochu D , Van Dyk J . A quality assurance phantom for three‐dimensional radiation therapy treatment planning. Int J Radiat Oncol Biol Phys. 1999;44(4):955–66. 10386655 10.1016/s0360-3016(99)00070-x

[17] Pooler AM , Mayles HM , Naismith OF , Sage JP , Dearnaley DP . Evaluation of margining algorithms in commercial treatment planning systems. Radiother Oncol. 2008;86(1):43–47. 18054103 10.1016/j.radonc.2007.11.006

[18] Stereoscopic Displays and Virtual Reality Systems XIII. Proc. of the SPIE Conference 2006 Jan 16–19, San Jose, California. SPIE Volume 6055.

[19] Schlaefer A , Blanck O , Shiomi H , Schweikard A . Radiosurgery: identification of efficient treatment beams guided by autostereoscopic visualization. GMS CURAC. 2006;1:Doc 14.

[20] Schlaefer A , Blanck O , Schweikard A . Autostereoscopic display of the 3D dose distribution to assess beam placement for robotic radiosurgery. Med Phys. 2005;32(6):2122.

[21] Shang C , Williams T , Beavis A. et al. Can current prostate IMRT be further improved with immersive virtual reality simulation? Med Phys. 2006;33:2075–76.

